# Cord blood hematopoietic cells from preterm infants display altered DNA methylation patterns

**DOI:** 10.1186/s13148-017-0339-1

**Published:** 2017-04-20

**Authors:** Olivia M. de Goede, Pascal M. Lavoie, Wendy P. Robinson

**Affiliations:** 1 BC Children’s Hospital Research Institute, Room 2082, 950W 28th Avenue, Vancouver, BC V5Z 4H4 Canada; 2 Department of Medical Genetics, University of British Columbia, Vancouver, BC V6T 1Z3 Canada; 3 Department of Pediatrics, University of British Columbia, Vancouver, BC V6T 1Z3 Canada

**Keywords:** DNA methylation, Cord blood, Preterm birth, Illumina 450K array, Epigenetics, Nucleated red blood cells, Epigenetic clock, Gestational age

## Abstract

**Background:**

Premature infants are highly vulnerable to infection. This is partly attributable to the preterm immune system, which differs from that of the term neonate in cell composition and function. Multiple studies have found differential DNA methylation (DNAm) between preterm and term infants’ cord blood; however, interpretation of these studies is limited by the confounding factor of blood cell composition. This study evaluates the epigenetic impact of preterm birth in isolated hematopoietic cell populations, reducing the concern of cell composition differences.

**Methods:**

Genome-wide DNAm was measured using the Illumina 450K array in T cells, monocytes, granulocytes, and nucleated red blood cells (nRBCs) isolated from cord blood of 5 term and 5 preterm (<31 weeks gestational age) newborns. DNAm of hematopoietic cells was compared globally across the 450K array and through site-specific linear modeling.

**Results:**

Nucleated red blood cells (nRBCs) showed the most extensive changes in DNAm, with 9258 differentially methylated (DM) sites (FDR < 5%, |Δβ| > 0.10) discovered between preterm and term infants compared to the <1000 prematurity-DM sites identified in white blood cell populations. The direction of DNAm change with gestational age at these prematurity-DM sites followed known patterns of hematopoietic differentiation, suggesting that term hematopoietic cell populations are more epigenetically mature than their preterm counterparts. Consistent shifts in DNAm between preterm and term cells were observed at 25 CpG sites, with many of these sites located in genes involved in growth and proliferation, hematopoietic lineage commitment, and the cytoskeleton. DNAm in preterm and term hematopoietic cells conformed to previously identified DNAm signatures of fetal liver and bone marrow, respectively.

**Conclusions:**

This study presents the first genome-wide mapping of epigenetic differences in hematopoietic cells across the late gestational period. DNAm differences in hematopoietic cells between term and <31 weeks were consistent with the hematopoietic origin of these cells during ontogeny, reflecting an important role of DNAm in their regulation. Due to the limited sample size and the high coincidence of prematurity and multiple births, the relationship between cause of preterm birth and DNAm could not be evaluated. These findings highlight gene regulatory mechanisms at both cell-specific and systemic levels that may be involved in fetal immune system maturation.

**Electronic supplementary material:**

The online version of this article (doi:10.1186/s13148-017-0339-1) contains supplementary material, which is available to authorized users.

## Background

Preterm birth (PTB), defined as birth prior to 37 weeks gestational age (GA), occurs in approximately 11% of live births and accounts for over half of infant mortality cases worldwide [1]. If a premature infant survives the immediate postnatal period, they face increased risk of developing major short- and long-term health problems including cerebral palsy, chronic lung disease, visual and hearing impairments, and adult metabolic diseases [2–6]. This elevated risk is attributable to organ immaturity, as well as an increased risk of medical complications linked to oxidative stress and inflammation during the neonatal period [7–9].

The immune system is not spared from the effects of PTB. The composition and function of hematopoietic cell populations change dramatically throughout gestation as the embryonic and fetal immune system mature. Premature interruption of the immunologically protected intrauterine environment results in an extremely fragile infant whose immune system is unprepared for the microbe-ridden external environment. A variety of systemic and cell-specific alterations in immune function have been identified in preterm infants that greatly increase their vulnerability to infection [10–12].

The importance of DNA methylation (DNAm) in the process of hematopoietic cell lineage commitment is well established [13, 14], and multiple studies have found differential methylation between cord blood of preterm and term infants [15–17]. However, these studies have used whole blood, which is a mixed-cell sample in which overall DNAm levels are influenced by cell composition [18, 19]. As a result, these studies cannot distinguish prematurity-associated DNAm patterns due to differences in cell composition from DNAm patterns reflecting developmental changes in immune function.

Using the Illumina Infinium Human Methylation 450 Bead Chip (450K array), we provide genome-wide DNAm profiles of T cells, monocytes, granulocytes, and nucleated red blood cells (nRBCs) collected from cord blood of infants born at term or highly preterm (<31 weeks GA). These DNAm profiles were compared between cell types and across GA to evaluate an epigenetic basis for altered neonatal immune function with prematurity. This study provides important insights into the role of DNAm in early hematopoietic system maturation in humans.

## Methods

### Study participants and sample collection

Ethics approval for this study was obtained from the University of British Columbia Children’s and Women’s (C&W) Research Ethics Board (certificate numbers H07-02681 and H04-70488). Written informed parental consent to participate was obtained. Individual patient data is not reported. Cord blood was collected from neonates delivered by caesarean section at the C&W Health Centre of BC (Vancouver, Canada). A total of 10 infants were involved in the study: 5 preterm (GA range 26–30 weeks) and 5 term (GA 38 weeks) (Table 1). None of the subjects had histological evidence of chorioamnionitis in the placenta. All term births were singleton, and the caesarean section was performed in the absence of labor. The preterm births had more variable clinical characteristics, including one case of preeclampsia, four births from multiple pregnancies, and a case of labor preceding the caesarean section (Table 1). In the cases of multiple pregnancies, only one subject was used and other siblings were excluded. Since the preterm births were all <31 weeks, immune function is expected to be significantly altered compared to term births regardless of the cause of prematurity.

**Table 1:** Subject characteristics and cell types collected from each subject

	Sex	GA (weeks)	Multiple birth	Presence of labor	Indication for PTB	Cells collected
term_1	M	38	No	No	n/a	all
term_2	M	38	No	No	n/a	all
term_3	F	38	No	No	n/a	all
term_4	M	38	No	No	n/a	all
term_5	M	38	No	No	n/a	all
preterm_A	M	26	No	No	Preeclampsia	T cells, nRBCs
preterm_B	F	29	Yes	No	Placental insufficiency	T cells, gran., mono.
preterm_C	M	30	Yes	No	Placental insufficiency	all
preterm_D	F	30	Yes	No	Placental insufficiency	T cells, mono., nRBCs
preterm_E	M	30	Yes	Yes	Twin-to-twin transfusion syndrome	all

For the column “Cells collected”: *all* T cells, granulocytes, monocytes, and nRBCs; *gran.* granulocytes; *mono.* monocytes; *n/a* not applicable

T cells, monocytes, and nRBCs were collected from cord blood by fluorescence-activated cell sorting (FACS). These sorting methods were designed to prevent erythrocyte-white blood cell (WBC) cross-contamination, a common occurrence in cord blood [20] and are described in detail in the Additional file 1. Granulocytes were collected by density gradient centrifugation and hypotonic red blood cell lysis. All cell populations were collected from all term subjects; however, due to small sample volumes and variability in blood cell counts, some cell populations could not be collected from some preterm subjects (Table 1).

### DNA extraction and DNA methylation data collection

DNA was extracted from all samples using standard protocols and purified with the DNeasy Blood & Tissue Kit (Qiagen, MD, USA). DNA was bisulphite-converted using the EZ DNA Methylation Kit (Zymo Research, CA, USA) before amplification and hybridization to the 450K array following manufacturer’s protocols (Illumina, CA, USA). Samples were randomly distributed across four 450K array chips, as shown in Additional file 1: Figure S1. 450K array chips were scanned with a HiScan reader (Illumina).

Raw intensity data for all hematopoietic cells were background corrected in GenomeStudio (Illumina). Quality control was performed using the 835 control probes included in the array. The intensity data were then exported from GenomeStudio and were converted into *M* values using the lumi package [21] in R software [22]. Sample identity and quality were evaluated as described in Additional file 1. The 450K array targets 485577 DNAm sites, but probe filtering was performed as described in Additional file 1 to produce a final dataset of 429765 sites. Red-green color bias was corrected for using the lumi package [21], and the data were normalized by subset within-array quantile normalization [23].

### DNA methylation data analysis

Unsupervised Euclidean clustering of the samples based on DNAm *β* values and principal component analysis based on DNAm *M* values were performed as exploratory global analysis steps. DNAm was then evaluated at subsets of the 450K array based on surrounding CpG density. These subsets are detailed in Additional file 1. Median DNAm (*β* values) of these CpG site groups were compared between all cell types using ANOVA followed by Tukey’s honest significant difference test, using a multiple comparison-adjusted *p* value threshold of 0.005. DNAm-based estimates of GA for the samples were calculated using a method developed by Knight et al. [24] in cord blood.

Differential methylation based on cell type and birth group (preterm or term) was assessed by linear modeling using the R package limma [25]. The same model was used to assess both PTB-associated and cell type-specific DNAm: the interaction of cell type and birth group was the variable of interest, and sex was included in the model as a covariate. Since each cell type was collected from the same set of individuals and the sample size was small, DNAm may have been influenced by inter-individual differences. To adjust for this, the model included a within-individual consensus correlation estimated using the *duplicateCorrelation()* function in limma [25]. Resulting *p* values were adjusted for multiple comparisons by the Benjamini and Hochberg [26] false detection rate (FDR) method.

For the comparison between preterm and term samples, statistically significant sites (“prematurity-associated DM sites”) were limited to those with an FDR < 5% and a |Δβ| > 0.10. Prematurity-associated DM sites were identified separately for each cell population. For cell type-specific DNAm, statistically significant sites (“cell type-DM sites”) were limited to those with an FDR < 5% and a |Δβ| > 0.20. Cell-type DM sites were identified separately within the two birth groups. ErmineJ was used to evaluate enrichment of gene ontology (GO) terms in genes associated with the cell-type and prematurity-associated DM sites [27].

Several other studies have performed similar evaluations of DNAm differences between preterm and term births, using whole cord blood instead of isolated cell populations [15–17]. The PTB-associated CpG sites discovered in those studies (29 CpG sites from Parets et al. [17]; 1347 CpG sites from Fernando et al. [16]; and 1555 CpG sites from Cruickshank et al. [15]) were overlapped with the prematurity-associated and the cell-type DM CpG sites identified in this study. Several subject characteristics varied between these studies, including cause of prematurity, ethnicity, and maternal age range. Overlapping sites are thus likely to be those present in multiple cell populations and also be unrelated to ethnicity or to PTB etiology. The specifics of these overlaps are described in Additional file 1.

To assess how prematurity-associated DNAm might reflect hematopoietic origin, DNAm patterns were compared between the birth groups and the cell types at a set of previously identified CpG sites that showed differential methylation between erythroblasts derived from fetal liver and erythroblasts derived from adult bone marrow (“source-DM sites”) [28]. These source-DM sites were divided into two groups: the top 100 CpG sites hypomethylated in adult BM erythroblasts (“BM-hypomethylated sites”) and the top 100 CpG sites hypomethylated in FL erythroblasts (“FL-hypomethylated sites”), with ranking and selection based on Lessard et al.’s *β* values. Median DNAm (*β* value) at these source-DM sites was compared between cell types and birth groups by ANOVA followed by Tukey’s honest significant difference test, with a multiple comparison-adjusted *p* value threshold of 0.005.

## Results

### Global DNA methylation profiles of preterm and term hematopoietic cells

**Fig. 1:**
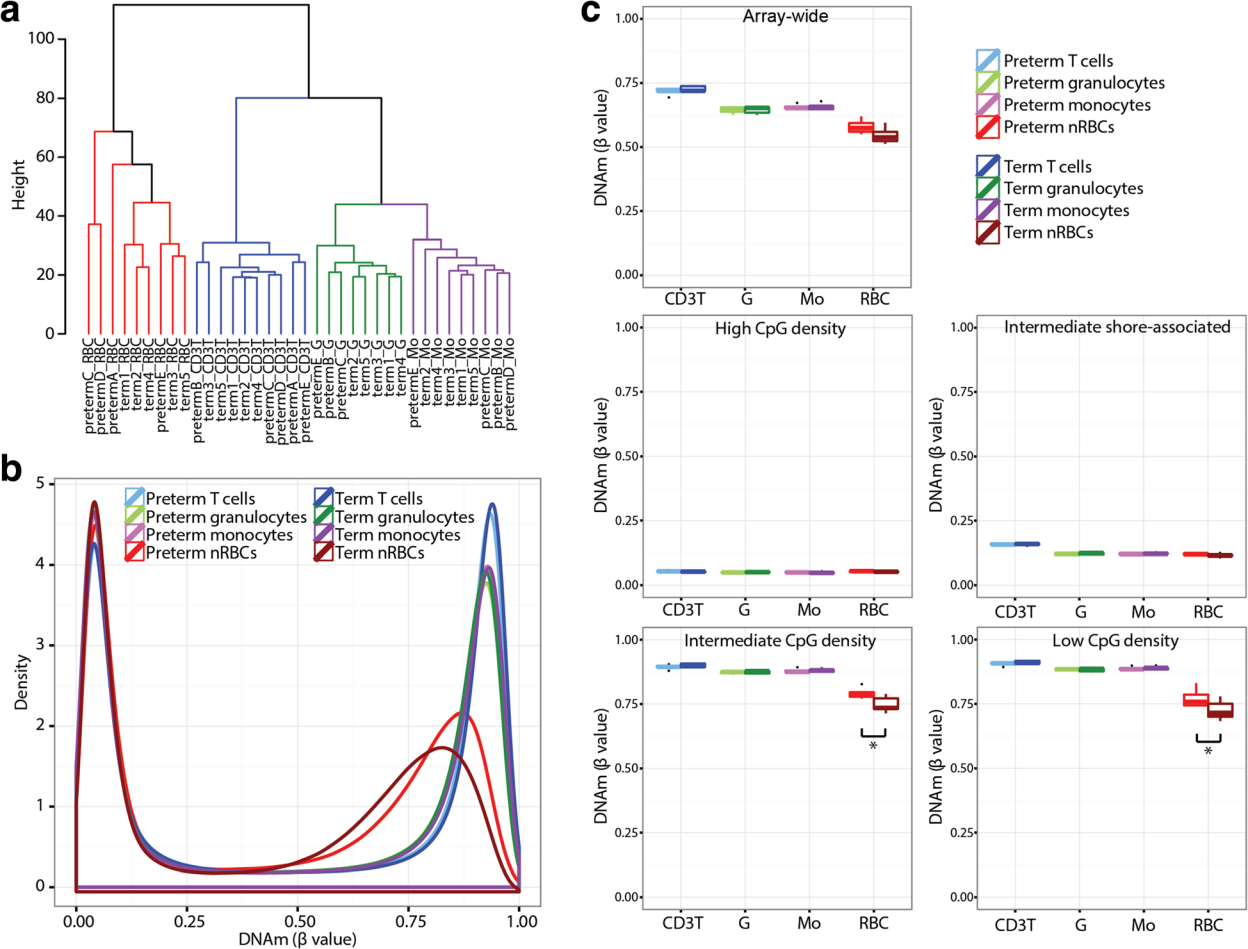
Genome-wide DNAm comparisons between term and preterm hematopoietic cells. **a** 450K array-wide Euclidean clustering of DNAm data for each preterm and term subject. **b** DNAm *β* value density distributions; each *line* represents the mean of that birth group/cell type combination. **c** Comparison of median DNAm between GA groups and cell types array-wide, and in CpG sites grouped by CpG density. *p < 0.05, **p < 0.005

Cell type was the dominant influence when DNAm profiles of term and preterm cell populations were compared by array-wide Euclidean clustering (Fig. 1a). Prematurity also had an observable impact on epigenetic relationships between the samples, with some samples clustering by birth group (preterm or term) within each cell type. However, these GA subgroups were not perfect, with some preterm samples clustering more closely with their term counterparts. Evaluating genome-wide DNAm by *β* value density distributions suggests that the effect of prematurity is largest in nRBCs. Term nRBCs were hypomethylated relative to preterm nRBCs, whereas all of the WBC populations showed similar distributions between term and preterm samples (Fig. 1b).

None of the cord blood hematopoietic cell populations differed in median array-wide DNAm between preterm and term infants; although term nRBCs were notably hypomethylated compared to preterm nRBCs, this difference was not significant (Fig. 1c). To identify genomic regions where the association between DNAm and prematurity is strongest, subsets of the 450K array based on CpG density were evaluated. In WBCs, no significant differences were observed between preterm and term samples at any of the CpG density subgroups. In nRBCs, term cells displayed hypomethylation relative to their preterm counterparts at the intermediate and low CpG density regions, however, these differences did not pass the multiple-test corrected significance threshold (*p* > 0.005).

### Prematurity-associated differentially methylated CpG sites

**Fig. 2:**
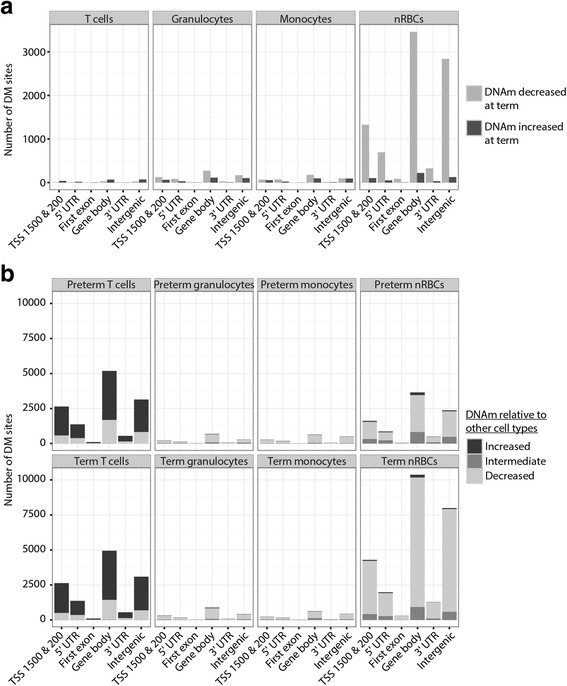
Prematurity-associated and cell type-DM sites grouped by gene region and changes in DNAm. **a** Prematurity-associated DM sites (FDR < 5%, |Δβ| > 0.10). **b** Cell type-DM sites (FDR < 5%, |Δβ| > 0.20). *TSS 1500 & 200* 1500 or 200 bp upstream from transcriptional start site, *UTR* untranslated region

Linear modeling was performed within each cell type to identify cell-specific prematurity-associated DM sites (FDR < 5%, |Δβ| > 0.10). nRBCs showed the greatest difference between preterm and term samples, with 9258 prematurity-associated DM sites; more than tenfold greater than observed in granulocytes, monocytes, and T cells (Table 2; Additional file 2). The majority of prematurity-associated DM sites were specific to a single cell type, making it unlikely that these changes were driven by chance genetic differences between the samples (Additional file 1: Figure S2). Twenty-five of the prematurity-associated DM sites were identified across all cell types, 17 of which increased in DNAm and 8 of which decreased in DNAm with GA (Additional file 2). All cell populations had the highest number of their prematurity-DM sites in the gene body and intergenic regions (Fig. 2a), which is consistent with the representation of these gene regions on the 450K array (33 and 24% of CpG sites assayed, respectively). In nRBCs, the TSS-upstream and 5′ UTR gene regions were also highly represented in prematurity-DM sites. This likely reflects the global nature of erythrocyte demethylation with maturity [29, 30].

**Table 2:** Number of prematurity-associated DM sites for each cell type (FDR < 5%, |Δβ| > 0.10)

	T cells	Granulocytes	Monocytes	nRBCs
Total	273	987	692	9258
DNAm decreases with GA	76 (28%)	679 (69%)	425 (61%)	8731 (94%)
DNAm increases with GA	197 (72%)	308 (31%)	267 (39%)	527 (6%)

The direction of DNAm change at prematurity-associated DM sites in each cell type paralleled their patterns of DNAm upon terminal differentiation (Table 2). For T cells, the high percentage of prematurity-associated DM sites with increased DNAm in term samples (72%) is consistent with increased DNAm with terminal differentiation of these cells [13, 14, 31]. For granulocytes and monocytes, the majority of prematurity-associated DM sites were hypomethylated in term samples (69 and 61%, respectively) in keeping with the documented loss of DNAm in myeloid cells [13, 14, 31]. For nRBCs, the vast majority of prematurity-associated DM sites (94%) showed reduced DNAm in term samples. Given that terminal erythroid differentiation is associated with global demethylation [29, 30], this change likely reflects an increasing proportion of mature erythroblasts in the nRBC population at term. Overall, these data suggest an epigenetic basis for the increased maturity of cord blood hematopoietic cell populations during fetal development.

GO pathway analysis of the prematurity-associated DM sites revealed enrichment of distinct sets of genes for each cell type (Additional file 3). The two significant GO terms in granulocytes (FDR < 10%) related to negative regulation of the ERK1 and ERK2 cascades, and Ras guanyl-nucleotide exchange factor activity. In T cells, the only significant GO term (FDR < 10%) was embryonic placenta development. Monocyte prematurity-associated DM sites were associated with eight significant GO terms (FDR < 10%), all of which were related to epidermal and hair growth and development. Evaluating the nRBC prematurity-associated DM sites revealed 152 significantly enriched GO terms (FDR < 10%); recurring themes in this list included Ras- and Rho-related activity, the cytoskeleton, and terms related to renal, muscle, and neuronal processes.

### Cell-specific DNA methylation patterns differ between preterm and term infants

After establishing the cell-specific DNAm differences between preterm and term births, we next investigated whether prematurity affects cell-type differences in DNAm (Additional file 4). Linear modeling revealed that nRBCs were the most distinct cell type in term samples, consistent with our previous findings [20, 32], but interestingly, T cells were the most distinct cell type of the preterm samples (Table 3). The relatively low number of monocyte- and granulocyte-DM sites in both GA groups was likely because these cell types are both of the myeloid lineage and thus epigenetically similar, in contrast to T cells and nRBCs, which are the only representatives of their respective hematopoietic lineages. In the WBC populations, the number of cell type-DM sites did not change drastically between preterm and term samples (Table 3). In contrast, the number of nRBC-DM sites nearly tripled between the preterm and term samples. This large change coincides with the increased hypomethylation in term nRBCs relative to their preterm counterparts (Fig. 1b), which made term nRBCs more distinct from term WBCs.

**Table 3:** Number of cell type-DM sites (FDR < 5%, | Δβ| > 0.20)

	T cells	Granulocytes	Monocytes	nRBCs
Preterm	12974	1410	1665	9056
Term	12662	1900	1508	26176
Common	10991 (85%, 87%)	1201 (85%, 63%)	1221 (73%, 81%)	7645 (84%, 29%)

Percentages of cell type-DM sites in common between the two GA groups are reported relative to the number of preterm DM sites first, then number of term DM sites

When the cell-type DM sites were compared between preterm and term samples based on gene region and the cell type of interest’s relative DNAm—that is, whether the unique cell population has DNAm that is higher, lower, or in between the DNAm levels of the other cell types—there was little difference in genomic representation or direction of DNAm change, particularly within WBCs (Fig. 2b). In nRBCs, the increase in the number of CpG sites hypomethylated relative to WBCs occurred in all gene regions, but most dramatically in the gene body and intergenic regions. Overall, this indicates that prematurity does not have a major impact on WBCs’ epigenetic relationships to each other. In contrast, nRBCs become more epigenetically distinct from WBCs as gestation progresses, adopting their uniquely hypomethylated profile [20, 32, 33]. The representation of these changes across all gene regions is likely reflective of nRBC demethylation being a global and largely passive process [29, 30].

### Comparison to other epigenetic studies of preterm birth

Previous studies have also identified distinctive DNAm patterns between preterm and term infants [15–17]. However, these studies were performed on either whole cord blood samples or the buffy coat and were not able to distinguish systematic prematurity-associated changes from those caused by shifts in cell composition across gestation. We compared our prematurity-associated and cell type-DM sites with the CpG sites found to be significantly associated with PTB (FDR < 5%) by Parets et al. [17] (29 CpG sites), Fernando et al. [16] (1347 CpG sites), and Cruickshank et al. [15] (1555 CpG sites).

**Fig. 3:**
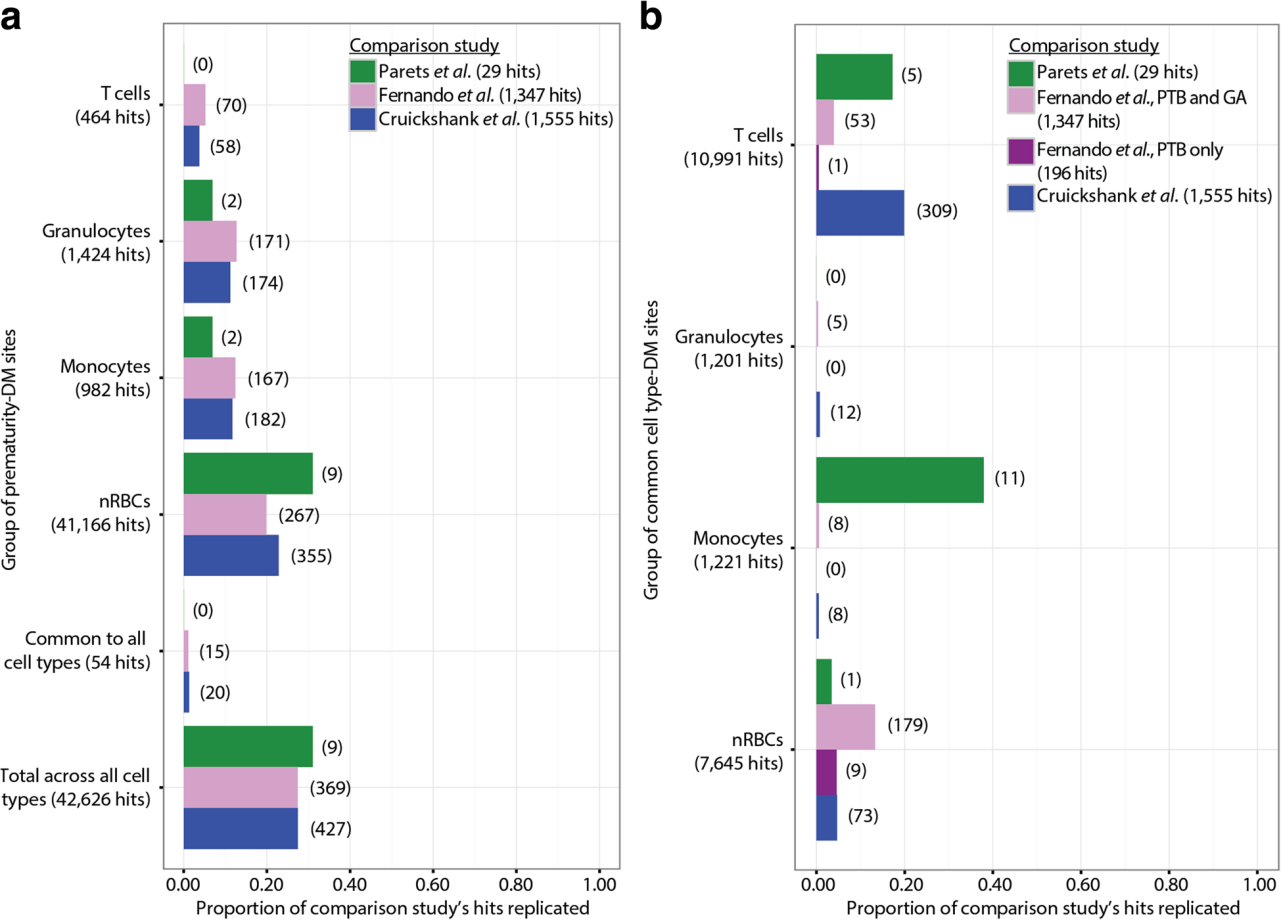
Overlap of prematurity-associated and cell type-DM sites with prematurity-associated CpG sites identified in previous studies. Proportion of prematurity-associated CpG sites found by Cruickshank et al., Fernando et al., and Parets et al. [15–17] also represented in (**a**) the prematurity-associated DM sites (FDR < 5%); and (**b**) the cell type-DM sites (FDR < 5%, |Δβ| > 0.20) identified in this study. The *numbers* beside *bars* are the number of overlapping CpG sites between the two lists

For all three of the comparison studies, approximately 30% of their DM sites were also discovered in at least one of our sets of prematurity-associated DM sites (9/29, 369/1347, and 427/1555 replicated DM sites) (Fig. 3a). When our identified cell type-DM sites were overlapped with the three comparison studies, the overlap was much lower than that with the prematurity-associated DM sites (Fig. 3b). The greatest overlap by number of sites occurred at T cell-specific and nRBC-specific DM sites (Fig. 3b). However, a notable proportion of Parets et al.’s [17] 29 differentially methylated CpG sites in PTB were associated with monocyte-specific DNAm in our data, a trend not seen in comparison with the other two studies. This could be due to a chance difference in average monocyte proportions between their preterm and term subjects or it could have come from Parets et al.’s [17] use of the buffy coat rather than the whole blood. Additionally, a subset of 196 of Fernando et al.’s [16] prematurity-associated CpG sites that were associated only with the state of being premature, and not with GA, showed almost no overlap with our cell type-DM sites (Fig. 3b).

A CpG site in *MYL4*, encoding myosin light chain 4, was the only DM site identified by Fernando et al. [16], Cruickshank et al*.* [15], and Schroeder et al. [34]; however, it was not observed in any of our cell populations. We also did not find any prematurity-associated DM sites in *ESR1*, encoding the estrogen receptor, in any of our cell populations, despite this gene being identified by both Fernando et al. [16] and Schroeder et al. [34]. However, we did replicate some of Fernando et al.’s [16] top findings of differential methylation in *NCOR2*, *DNAJC17*, *PYCR2*, *ATP6V0A1*, *RARA*, *FBLN7*, *IGF2BP1*, and *ATP2B2*, as well as differential methylation observed by Cruickshank et al. [15] in *NFIX*, *OXT*, *DNMT3A*, *RUNX1*, and *AIRE*. We also found prematurity-associated DNAm in *ADORA2A* and *GABBR1*, which was identified by both of these studies. Of the 54 prematurity-associated DM sites we observed across all cell types, seven were also identified by both Fernando et al. [16] and Cruickshank et al. [15]. Of these shared CpG sites, two are located in the gene body of *WWTR1*, and two are located in the 5′UTR of *CLIP2*; the other three are intergenic.

**Fig. 4:**
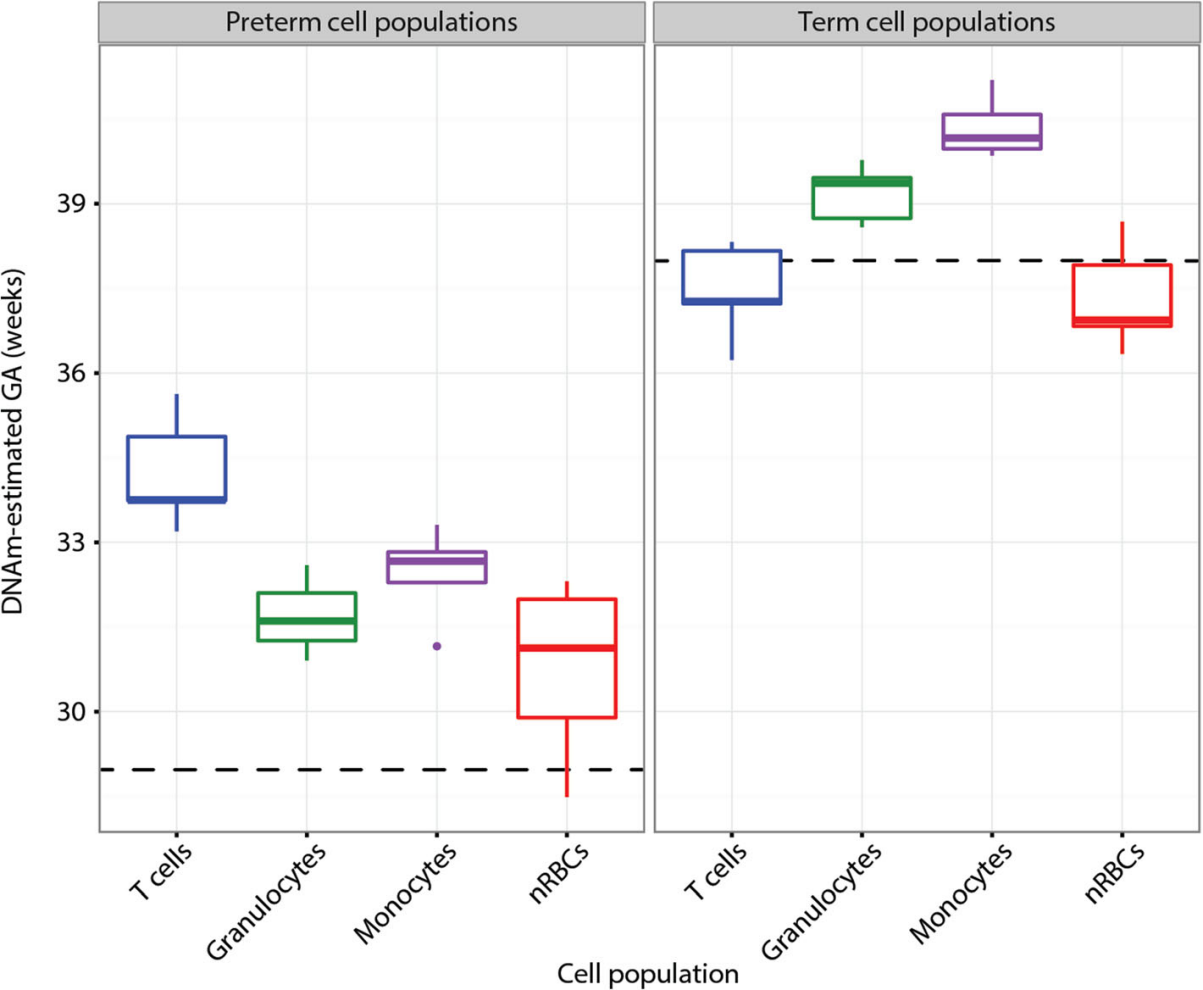
DNAm-based estimates of gestational age (GA) by cell type and birth group. *Dashed lines* reflect mean actual GA for the birth group. Estimates calculated using methods published by Knight et al. [24]

To further explore how the cell-specific DNAm changes we observed compared to trends in whole cord blood, we applied the recently published epigenetic clock for GA to our data [24]. This GA-epigenetic clock was designed using cord blood samples and, unlike the epigenetic clock designed for adult samples [35], was only validated in cord blood. This is unsurprising, since cord blood is the most frequently studied tissue in studies of the fetus or neonate. However, we were curious to see how this whole blood-based algorithm would perform on its constituent cell types. In all preterm cell populations, estimated DNAm GA was an overestimate of actual GA (Additional file 5). In term samples, the GA estimates were more accurate: when estimated GA was averaged across all cell types within an individual, none of the term individuals had estimates over 1 week different than their actual GA (Additional file 5). There were also some intriguing cell type-specific trends in GA estimates: for example, T cells had the highest GA estimates in preterm individuals, but one of the lowest in term individuals (Fig. 4). Additionally, monocytes in term individuals were consistently estimated as the “oldest” cell population, whereas nRBCs had low GA estimates regardless of birth group.

### DNA methylation associated with prematurity may reflect hematopoietic origin

**Fig. 5:**
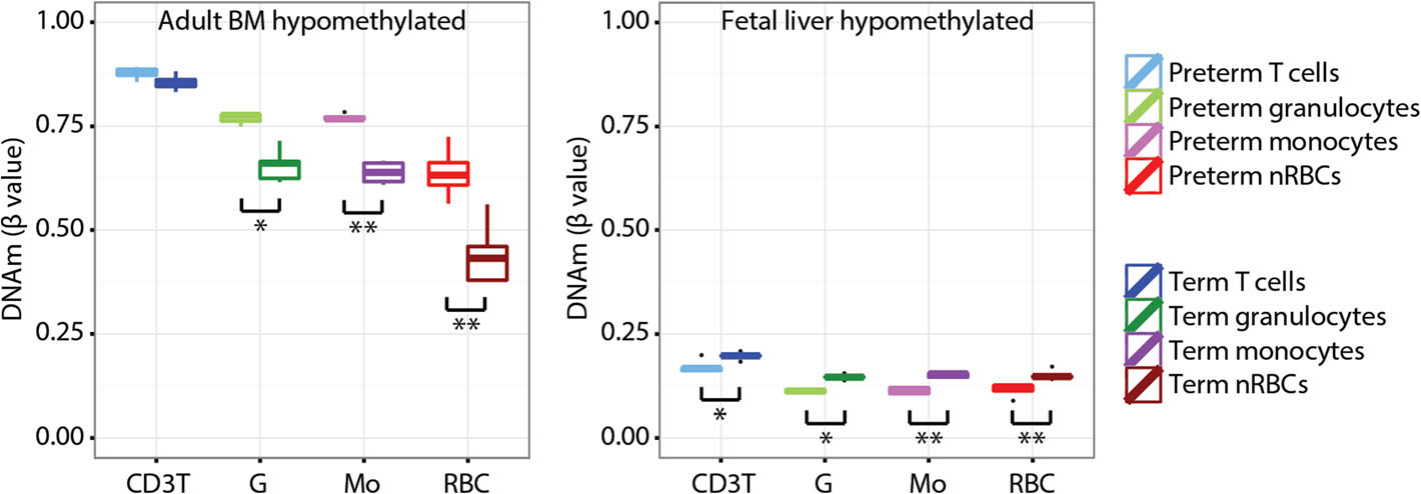
Comparison of median DNAm between GA groups and cell types at CpG sites associated with hematopoietic origin. DNAm was compared at the top 100 CpG sites hypomethylated in erythroblasts derived from adult bone marrow stem cells (*left*) and the top 100 CpG sites hypomethylated in erythroblasts derived from fetal liver stem cells, as identified by Lessard et al. [28]. *p < 0.05, **p < 0.005

While changes in DNAm may in part reflect an aging “clock” [24], our cell-specific GA-epigenetic clock analyses above suggest that other factors can modify this trend. One such factor may be the predominant hematopoietic organ, which shifts from the liver to the bone marrow early in the third trimester of gestation [36]. We hypothesized that the preterm samples used in this study, which range from 26 to 30 weeks GA, have a greater proportion of liver-derived cells than the term samples. Hematopoietic source-related methylation differences with PTB were evaluated using CpG sites previously associated with liver- or bone marrow-specific DNAm in ex vivo-derived nRBCs [28]. From these 5937 “source-DM sites”, two groups of CpG sites were assessed: the top 100 sites that were hypomethylated in adult bone marrow-derived nRBCs relative to fetal liver-derived nRBCs (“BM-hypomethylated sites”) and the top 100 sites that displayed the opposite pattern (“FL-hypomethylated sites”). Only one of these 200 CpG sites overlapped with the 148 CpG sites used in the GA-epigenetic clock [24]. In our samples, all cell types displayed the same trend, with preterm samples less methylated at FL-hypomethylated sites and term samples less methylated at BM-hypomethylated sites (Fig. 5). This difference at BM-hypomethylated sites was significant in all cell types except T cells.

Overlapping our prematurity-associated DM sites with Lessard et al.’s hematopoietic source-DM sites provided further support for a relationship between DNAm and hematopoietic origin of cord blood cell types (Table 4). CpG sites that increased in DNAm with increasing GA overlapped almost exclusively with fetal liver-hypomethylated CpG sites, likely reflecting the reduced contribution of the liver to hematopoiesis as gestation progresses. In contrast, many of the CpG sites that decreased in DNAm with increasing GA were also associated with hypomethylation in bone marrow-derived hematopoietic cells, corresponding with this organ becoming the primary source of hematopoietic cells towards the end of the third trimester.

**Table 4:** Overlap between cell-specific prematurity-associated DM sites (FDR < 5%, |Δβ| > 0.10) and Lessard et al.’s source-DM sites [25]

	DNAm decreases with GA				DNAm increases with GA			
	T cell	Gran.	Mono.	nRBC	T cell	Gran.	Mono.	nRBC
Total	76	679	425	8731	197	308	267	527
Overlap with BM-hypo. sites	25, 32.9%	197, 29.0%	213, 50.1%	895, 10.3%	1, 0.5%	0, 0.0%	1, 0.4%	1, .02%
Overlap with FL-hypo. sites	0, 0.0%	1, 0.1%	0, 0.0%	2, 0.0%	22, 11.2%	74, 24.0%	70, 26.2%	89, 16.9%

*Gran.* granulocytes, *mono.* monocytes

## Discussion

Previous DNAm studies using cord blood have identified significant differences between preterm and term infants [15–17]; however, interpretation of these studies is limited by the confounding factor of cord blood cell composition. Granulocytes and T cells are the two most abundant cell types in whole blood and thus are the most likely to influence overall DNAm, but cell type proportions show considerable inter-individual variability and also change with gestational age [12]. Some DNAm changes previously associated with prematurity may simply reflect these changes in cell composition with GA. This study is the first evaluation of the epigenetic impact of PTB in hematopoietic cell populations isolated from the same individuals.

An important question is the functional role of these DNAm changes in hematopoietic cell populations during ontogeny. There was a notable difference in the number of cell-specific prematurity-associated DM sites in each cell type, ranging from 273 in T cells to 9258 in nRBCs (Table 2). The number of prematurity-associated DM sites in a given cell type may relate to the magnitude of phenotypic differences between preterm and term populations. For example, we and others have reported major functional differences in dendritic cells and macrophages between preterm and term infants [37–40]. In contrast, fewer functional differences have been observed between preterm and term T cells [41, 42]. For granulocytes, much less is known regarding gestational differences. The high number of prematurity-associated DM sites we observed in granulocytes (987) suggests a more dynamic maturation across late gestation than for either monocytes or T cells. Alternatively, it is possible that these DNAm changes may reflect differences in the composition of granulocyte subsets, including a mixture of eosinophils, basophils, and mast cells, between age groups. However, this is less likely given that our granulocytes were overwhelmingly represented by neutrophils in both preterm and term samples (>95%; data not shown). Given the extent of DNAm differences between preterm and term granulocytes, functional studies may provide new insight into the limitations of the preterm immune system.

Our findings showed moderate overlap with previous studies of prematurity-associated DNAm in whole cord blood, with approximately 30% of the DM sites from each comparison study also discovered in at least one of our cell types (Fig. 3a). This is a greater amount of overlap than the 161 of 1347 CpG sites Fernando et al. [16] found in common with Cruickshank et al. [15] and another study not evaluated in this paper [34]. This increased overlap with other studies may reflect reduced noise our data due to eliminating variation due to cell composition differences. Alternatively, since we compared four sets of prematurity-associated DM sites (one per cell type), all of which were of fairly large size, we may have increased our chance of overlap just by having a greater number of hits.

When the cell type-DM sites discovered in this study are overlapped with the three comparison studies, the highest numbers of overlapping sites were observed with T cell- and nRBC-specific DM sites, the two cell types with the strongest cell-specific DNAm patterns (Fig. 3b). We additionally compared our cell type-DM sites to a subgroup of 196 CpG sites identified by Fernando et al. to be associated with PTB but not GA—and thus thought to reflect systematic differences due to prematurity rather than cell composition—and found almost no overlap (Fig. 3b). This supports their assertion that those 196 CpG sites are more likely related to the molecular mechanisms of PTB than cell composition, compared to the 1151 CpG sites they identified as associated with both PTB and GA. Thus, subsequent studies of PTB may be able to work around concerns of variability in cell composition by considering prematurity as a separate variable from GA.

Two CpG sites highlighted by Fernando et al. as being of potential interest for preterm delivery were not replicated in our study. One site, in *MYL4* (myosin light chain 4), was the only DM site identified by Fernando et al. [16], Cruickshank et al. [15], and Schroeder et al. [34]; the other, *ESR1* (estrogen receptor) was observed in both Fernando et al. [16] and Schroeder et al. [34]. Fernando et al. suggested that these sites may be related to the labor process, since *MYL4* activity is involved in the myometrial contraction pathway [43], and upregulation of *ESR1* leads to the increase in estrogen activity required for contractions [44]. The lack of replication in our data may be a consequence of all of our subjects being born by caesarean section, whereas all of the comparison studies included at least some subjects born by vaginal delivery. Notable genes in which we replicated the differential methylation found by other studies include *ADORA2A*, which has been associated with the inflammatory pathway in the myometrium [45], and *GABBR1*, which encodes a gamma-aminobutyric acid receptor and has been associated with chemotaxis in cord blood-derived stem cells [46]. These two genes were identified as differentially methylated in both Fernando et al. [16] and Cruickshank et al. [15].

GO pathway analyses of the prematurity-associated DM sites highlighted potentially important differences in gene regulation that are unique to each cell type (Additional file 3). For instance in granulocytes, prematurity-associated DM sites were significantly enriched for genes associated with the Ras-Raf-MEK-ERK cascade. Defects in this pathway have been associated with impaired neutrophil extracellular trap formation and with respiratory burst in neutrophils [47, 48], both of which are also deficient in preterm infants [49, 50]. The prevalence of prematurity-associated DM sites in genes associated with these functions could reflect either reduced functional ability in preterm neutrophils or a low proportion of neutrophils within the preterm granulocyte population. Some of our findings point towards novel pathways potentially involved in the maturation of hematopoietic cells, such as the enrichment for prematurity-associated DM sites in genes associated with placental development in T cells and with dermal development in monocytes. In nRBCs, prematurity-associated DNAm changes were widespread and associated with GO terms related to the cytoskeleton, membrane composition and cell-cell junctions, and motility. This may reflect the large-scale structural changes that occur in erythroblasts as they mature and prepare to extrude their nucleus.

Based on gestational age differences, DNAm conformed to the epigenetic profile of the dominant hematopoietic organ when evaluated in source-DM sites [28]: the liver in mid-gestation, and the bone marrow in late gestation (Fig. 5). Despite these candidate sites being identified exclusively in nRBCs derived ex vivo from hematopoietic stem cells, the DNAm trends in this study were consistent across both nRBCs and WBCs. Thus, our findings indicate that hematopoietic sources have epigenetic signatures that are shared across multiple cell lineages derived from that organ. Additionally, our analysis of these hematopoietic source-DM sites revealed that nRBCs actually gain DNAm with increasing GA in functionally relevant regions of the genome, specifically the fetal liver-hypomethylated sites. This is a rare occurrence, as the overwhelming trend is for nRBCs to become demethylated both during erythropoiesis [29, 30] and as the fetus approaches term. This novel observation is important to our understanding of hematopoiesis during ontogeny since it indicates that although nRBC demethylation is largely global and passive [29, 30], it also has some selectivity, with certain CpG sites protected from the widespread DNAm loss.

The main limitation in our study is the small sample size. Other studies evaluating prematurity-associated DNAm had sample sizes ranging from 22 [16] to 50 [17]. With only ten subjects, our study had reduced power to detect changes in DNAm. Considering the large epigenetic differences between cell lineages, we expect that our study was sufficiently powered to compare cell types. However, differential methylation associated with prematurity is expected to be of a smaller scale than cell type differences, so this may have led to an underestimation of prematurity-associated DM sites. There was also an increased chance that genetic factors impacted our findings, since some CpG sites are methylation quantitative trait loci (mQTLs), or sites where DNAm is more strongly associated with individuals than cell type [20, 51]. We mitigated this concern by performing an additional probe filtering step to remove suspected mQTLs, as described in the Supplementary Methods (Additional file 1).

It is possible that heterogeneity in our subjects’ clinical characteristics reduced our ability to detect prematurity-associated differential methylation. All births were caesarean sections with no indications of infection; however, one preterm case was attributed to preeclampsia, and four of the five preterm births were multiples (Table 1). This raises the concern that multiplicity in the preterm subjects may have confounded our results. There is limited information on how the immune system differs with multiple births: it has been shown that intrauterine infection occurs more often in preterm births with dizygotic twins compared to monozygotic twins or singletons, but no differences in postnatal outcome have been associated with zygosity [52]. Additionally, CD4^+^ T cell activity has been observed to be significantly lower in preterm dizygotic twins than in preterm singletons [53]. For DNAm studies, the effect of twin births has only been assessed within twin pairs [54], not between twins and singletons. For our study, considering the extreme difference in GA of our preterm and term cases (<31 versus >38 weeks), we expect that prematurity will have a much greater effect on DNAm than differences due to multiplicity. This is in keeping with Fernando et al.’s [16] observation of more distinct clustering of DNAm in extreme PTB cases compared to intermediate PTB cases. Despite these limitations, we identified prematurity-DM sites that showed reasonable overlap with prior studies [15–17] and cell-specific DNAm patterns that were consistent with our previous findings in cord blood cell populations [20, 32].

## Conclusions

The preterm immune system differs from that of the term neonate in both cell composition and function, resulting in heightened vulnerability to infection in preterm infants. We identified epigenetic markers of immune system differences with prematurity by comparing DNAm of major cord blood hematopoietic cell populations across gestation. Changes in DNAm between preterm and term hematopoietic cells in our study likely reflect a shift from the liver to the bone marrow as the predominant hematopoietic source with advancing gestational age. Granulocytes were identified as a candidate cell population of particular interest in preterm infants’ susceptibility to infection, due to their relatively high number of prematurity-associated DM sites and the enrichment of these sites for GO terms related to the Ras-Raf-MEK-ERK cascade. Our findings provide important insights into the epigenetic regulation of hematopoietic cell-specific functions during fetal development. These data may have clinical implications, as they highlight gene regulatory mechanisms on both cell-specific and systemic levels that are involved in neonatal immune system maturity. Larger samples will be required to determine the potential impact of cause of PTB (such multiple gestations or preeclampsia) on these epigenetic profiles.

## Additional files

Additional file 1: Supplementary methods and figures. (DOCX 207 kb)

Additional file 2: “Location and genomic context of prematurity-DM sites”. Each sheet is a table summarizing prematurity-DM sites (FDR < 5%, |Δβ| > 0.10) for each cell type, as well as one for the DM sites common across all cell types. (XLSX 1890 kb)

Additional file 3: Significantly enriched GO terms (corrected *p* value <0.10) from ErmineJ analysis of prematurity-DM sites for each cell type, ordered by corrected *p* value. (XLSX 18 kb)

Additional file 4: “Location and genomic context of cell type-DM sites”. Each sheet is a table summarizing cell type-DM sites (FDR < 5%, |Δβ| > 0.20) for each birth group/cell type combination. (XLSX 11523 kb)

Additional file 5: DNAm-based estimates of gestational age (GA) using Knight et al.’s [24] methods. (XLSX 10 kb)

## Abbreviations

450K array: Illumina Infinium Human Methylation 450 BeadChip
BM: Bone marrow
DM: Differentially methylated
DNAm: DNA methylation
FDR: False detection rate
FL: Fetal liver
GA: Gestational age
GO: Gene ontology
nRBC: Nucleated red blood cell
PTB: Preterm birth
WBC: White blood cell
