# Supplementary material for: Cord blood hematopoietic cells from preterm infants display altered DNA methylation patterns
Source: Clin Epigenetics. 2017 Apr 20;9:39. doi: 10.1186/s13148-017-0339-1 (PMC5397745; doi:10.1186/s13148-017-0339-1)
Supplement: Supplementary file 1 — Supplementary methods and figures. (DOCX 207 kb) [file 13148_2017_339_MOESM1_ESM.docx]

Supplementary Methods

**Study participants and sample collection**

Ethics approval for this study was obtained from the University of British Columbia Children’s and Women’s (C&W) Research Ethics Board (certificate numbers H07-02681 and H04-70488). Written, informed parental consent to participate was obtained. Individual patient data is not reported. Cord blood was collected from neonates delivered by elective caesarean section at the Children’s and Women’s Health Centre of BC (Vancouver, Canada). Cells were sorted from a total of 10 subjects: 5 preterm (GA range 26-30 weeks) and 5 term (GA 38 weeks). Patient characteristics are summarized in Table 1.

Cord blood was collected in sodium heparin anti-coagulated Vacutainers (Becton Dickinson, ON, Canada). CBMCs were extracted using a Lymphoprep (StemCell Technologies Inc., BC, Canada) density gradient centrifugation, washed and resuspended in phosphate-buffered saline.

T cells, monocytes, and nRBCs were purified by FACS using a strategy designed to eliminate erythrocyte-white blood cell cross-contamination [de Goede *et al.*, 2015]. The following conjugated antibodies were used to sort these cells: anti-CD3 PE (clone UCHT1; BD Bioscience), anti-CD14 PE-Cy7 (clone 61D3; eBioscience), anti-CD19 Alexa Fluor 700 (clone HIB19; eBioscience), anti-CD71 APC (clone OKT9; eBioscience) and anti-CD235 FITC (clone 10F7MN; eBioscience). Unstained, single-stain compensation and FMO controls were prepared for each sample run. Compensation for spectral overlaps between fluorophores was done before cell acquisition. Cells were sorted on the FACSAria III flow cytometer using FACSDiva Software (both Becton Dickinson). Data analysis was performed with Flowjo software (TreeStar, Inc., OR, USA). T cells were sorted according to the following parameters: CD3+/CD19-/CD235-/CD14-. Monocytes were sorted according to the following parameters: CD3-/CD19-/CD14+/CD71-. nRBCs were sorted according to the following parameters: CD3-/CD19-/CD235+/CD71+.

Granulocytes were obtained from the bottom fraction of the Lymphoprep gradient during CBMC purification, mixed with 3% dextran/0.9% saline solution to allow separation of granulocytes from erythrocytes by sedimentation, and followed by three steps of hypotonic lysis. Hypotonic lysis was achieved by incubation of the granulocyte fraction with ice cold 0.2% sodium chloride (NaCl) for 30 seconds to lyse remaining red blood cells. Following lysis, isotonicity was restored by adding an equal volume of 1.6% NaCl solution at room temperature.

**DNA methylation data collection and quality control**

DNA was extracted from all samples using standard protocols and purified with the DNeasy Blood and Tissue kit (Qiagen, MD, USA). DNA was bisulphite-converted using the EZ DNA Methylation Kit (Zymo Research, CA, USA) before amplification and hybridization to the 450K array following manufacturer’s protocols (Illumina, CA, USA). Samples were randomly distributed across four 450K array chips, as shown in Supplementary Figure 1. 450K array chips were scanned with a HiScan reader (Illumina).

Raw intensity data for all hematopoietic cells were background corrected in GenomeStudio (Illumina). Quality control was performed using the 835 control probes included in the array. The intensity data were then exported from GenomeStudio and converted into M values using the lumi package [Du *et al.*, 2008] in R software [R Core Team, 2014]. Once 450K array intensity data were converted into M values in R software, sample identity and quality were evaluated in three ways: 1) clustering with the 65 SNP probes provided on the array, with samples from the same individual grouping together as expected; 2) clustering with probes on the X and Y chromosomes, with samples grouping by known sex as expected; 3) clustering based on all probes, producing groups based on cell type.

Probes were removed from analysis if they fell into any of the following categories: 1) probes that target SNPs (n = 65); 2) probes that target or cross-hybridize with sites on the sex chromosomes (n = 11,648 and 11,366, respectively); and 3) probes that target CpGs which may also contain SNPs (n = 19,283) [Price *et al.*, 2013]. Probes that had a detection p-value >0.01 or under 3 bead replicates in more than one sample were also removed (n = 2,943). Additional filtering was performed to remove CpG sites that are suspected mQTLs: for each probe, the DNAm standard deviations within each cell type and within each individual were calculated. If the mean cell type standard deviation was both greater than the mean individual standard deviation and greater than 0.05, that probe was removed from the dataset. 10,507 probes met these criteria and were filtered out, producing a final dataset of 429,765 probes. Red-green color bias was corrected for using the lumi package [Du *et al.*, 2008], and the data were normalized by subset within-array quantile normalization [Maksimovic *et al.*, 2012].

**DNA methylation data analysis**

Global trends in DNAm were evaluated by unsupervised Euclidean clustering with β values and principal component analysis with M values. Probes on the 450K array were then grouped based on CpG density and median DNAm (β value) was compared between the cell types at these regions using ANOVA followed by Tukey’s honest significant difference test with a multiple comparison-adjusted p-value threshold of 0.005. The groups of CpG sites by CpG density were as follows: 141,866 CpG sites in regions of high density; 30,369 CpG sites in CpG shore-associated regions of intermediate density; 101,122 CpG sites in regions of intermediate density; and 156,408 CpG sites in regions of low density. DNAm-based estimates of gestational age were produced using R scripts provided by Knight *et al.* (2016), who designed and validated this approach on whole cord blood.

Differential methylation based on cell type and birth group (preterm or term) was assessed by linear modelling using the R package limma [Ritchie *et al.*, 2015]. For all linear modelling analyses, the models were applied to DNAm M values, but filtering of significant CpG sites based on effect size (DNAm difference) was based on β values. Significant CpGs were also reported as β values for ease of interpretation.

To assess PTB-associated DNAm changes in each hematopoietic cell population, the combination of cell type and birth group was modelled as the variable of interest and sex was included in the model as a covariate. Since each cell type was collected from the same set of individuals, DNAm may have been influenced by inter-individual differences. To adjust for this, the model included a within-individual consensus correlation estimated using the *duplicateCorrelation()* function in limma [Ritchie *et al.*, 2015]. Resulting p-values were adjusted for multiple comparisons by the Benjamini & Hochberg (1995) false detection rate (FDR) method, and statistically significant sites (“prematurity-associated DM sites”) were limited to those with an FDR <5% and a |Δβ| >0.10.

To identify cell-type specific DNAm in the preterm and term immune system, linear modelling was performed with the same model described above. Resulting p-values were adjusted for multiple comparisons by the Benjamini & Hochberg (1995) FDR method, and statistically significant sites (“cell-type DM sites”) were limited to those with an FDR <5% and a |Δβ| >0.20. ErmineJ was used to evaluate enrichment of gene ontology (GO) terms in genes associated with the cell-type and prematurity-associated DM sites [Gillis *et al.*, 2010].

Several other studies have performed similar evaluations of DNAm differences between preterm and term births in whole cord blood [Cruickshank *et al.,* 2013; Fernando *et al.*, 2015; Parets *et al.*, 2013]. The PTB-associated CpG sites discovered in those studies (29 CpG sites from Parets *et al.*; 1,347 CpG sites from Fernando *et al.*; and 1,555 CpG sites from Cruickshank *et al.*) were overlapped with the prematurity-associated and the cell-type DM CpG sites identified in this study. Since all of the comparison studies used only a FDR threshold and did not additionally filter by DNAm difference, the prematurity-associated DM sites from this study were relaxed to just FDR <5% to perform these overlaps. This resulted in the following numbers of prematurity-associated DM sites: 464 CpG sites in T cells; 1,424 CpG sites in granulocytes; 982 CpG sites in monocytes; 41,166 CpG sites in nRBCs; and 54 CpG sites common to all four cell types. Overlapping was additionally done with this study’s cell-type DM sites that were identified in both birth groups (FDR <5%, |Δβ| >0.20): 10,991 T cell-specific CpG sites; 1,201 granulocyte-specific CpG sites; 1,221 monocyte-specific CpG sites; and 7,645 nRBC-specific CpG sites. The cell-type DM sites were also overlapped with a subset of 196 of Fernando *et al.*’s 1,347 DM sites that they found to be associated with PTB but not with GA. The proportions of overlap are reported relative to the number of CpG sites identified by the comparison study.

To assess how prematurity-associated DNAm might reflect hematopoietic origin, DNAm patterns were compared between the GA groups and cell types at a set of previously-identified CpG sites that showed differential methylation between erythroblasts derived from fetal liver and erythroblasts derived from adult bone marrow [Lessard *et al.*, 2015] (referred to in this paper as “source-DM sites”). These source-DM sites were divided into two groups: the top 100 CpG sites hypomethylated in adult BM erythroblasts (“BM-hypomethylated sites”), and the top 100 CpG sites hypomethylated in FL erythroblasts (“FL-hypomethylated sites”), with ranking and selection based on Lessard *et al.*’s β values. DNAm at these source-DM sites was compared between cell types and birth groups by ANOVA followed by Tukey’s honest significant difference test, with a multiple comparison-adjusted p-value threshold of 0.005.

References

Benjamini Y, Hochberg Y. Controlling the false discovery rate: A practical and powerful approach to multiple testing. Journal of the Royal Statistical Society. 1995;57:289-300.

Cruickshank MN, Oshlack A, Theda C, et al. Analysis of epigenetic changes in survivors of preterm birth reveals the effect of gestational age and evidence for a long term legacy. Genome Med. 2013;5(10):96.

de Goede OM, Razzaghian HR, Price EM, Jones MJ, Kobor MS, Robinson WP, et al. Nucleated red blood cells impact DNA methylation and expression analyses of cord blood hematopoietic cells. Clin Epigenetics. 2015;7(1):95.Du P, Kibbe WA, Lin SM. Lumi: A pipeline for processing Illumina microarray. Bioinformatics. 2008;24(13):1547-1548.

Du P, Kibbe WA, Lin SM. lumi: A pipeline for processing Illumina microarray. Bioinformatics. 2008;24(13):1547-1548.

Fernando F, Keijser R, Henneman P, van der Kevie-Kersemaekers AMF, Mannens MM, van der Post JAM, et al. The idiopathic preterm delivery methylation profile in umbilical cord blood DNA. BMC Genomics. 2015;16:736-015-1915-4.

Gillis J, Mistry M, Pavlidis P. Gene function analysis in complex data sets using ErmineJ. Nat Protoc. 2010;5(6):1148-1159.

Knight AK, Craig JM, Theda C, Bækvad-Hansen M, Bybjerg-Grauholm J, Hansen CS, et al. An epigenetic clock for gestational age at birth based on blood methylation data. Genome Biol. 2016;17:206.

Lessard S, Beaudoin M, Benkirane K, Lettre G. Comparison of DNA methylation profiles in human fetal and adult red blood cell progenitors. Genome Med. 2015;7(1):1-014-0122-2. eCollection 2015.

Maksimovic J, Gordon L, Oshlack A. SWAN: Subset-quantile within array normalization for Illumina Infinium HumanMethylation450 BeadChips. Genome Biol. 2012;13(6):R44-2012-13-6-r44.

Parets SE, Conneely KN, Kilaru V, Fortunato SJ, Syed TA, Saade G, et al. Fetal DNA methylation associates with early spontaneous preterm birth and gestational age. PLoS One. 2013;8(6):e67489.

Price ME, Cotton AM, Lam LL, et al. Additional annotation enhances potential for biologically-relevant analysis of the Illumina Infinium HumanMethylation450 BeadChip array. Epigenetics Chromatin. 2013;6(1):4-8935-6-4.

R Core Team. R: A language and environment for statistical computing. Vienna, Austria: the R Foundation for Statistical Computing; 2014.

Ritchie ME, Phipson B, Wu D, Hu Y, Law CW, Shi W, et al. limma powers differential expression analyses for RNA-sequencing and microarray studies. Nucleic Acids Res. 2015;43(7):e47.

Supplementary Figures


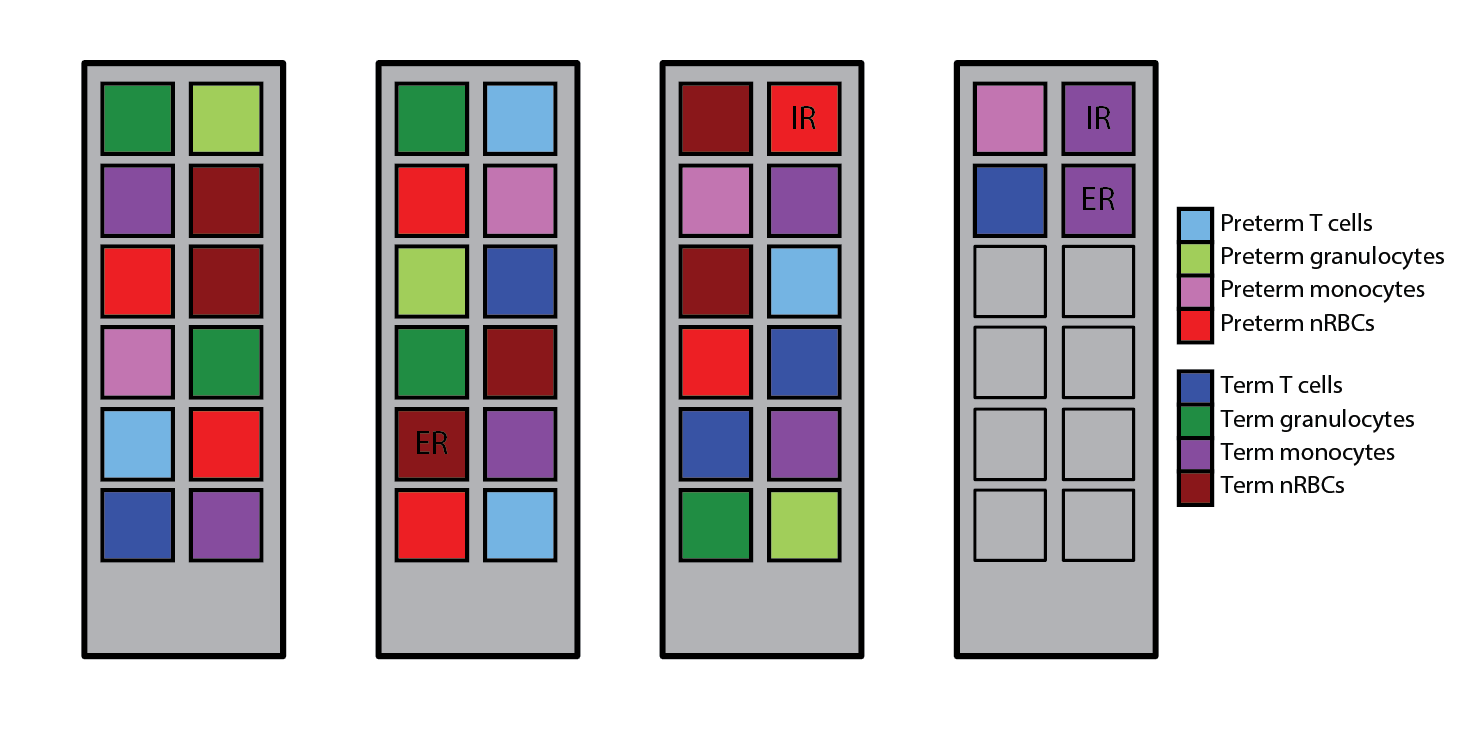


**Figure S1. Distribution of samples across 450K array chips.** IR = internal replicate; ER = external replicate.


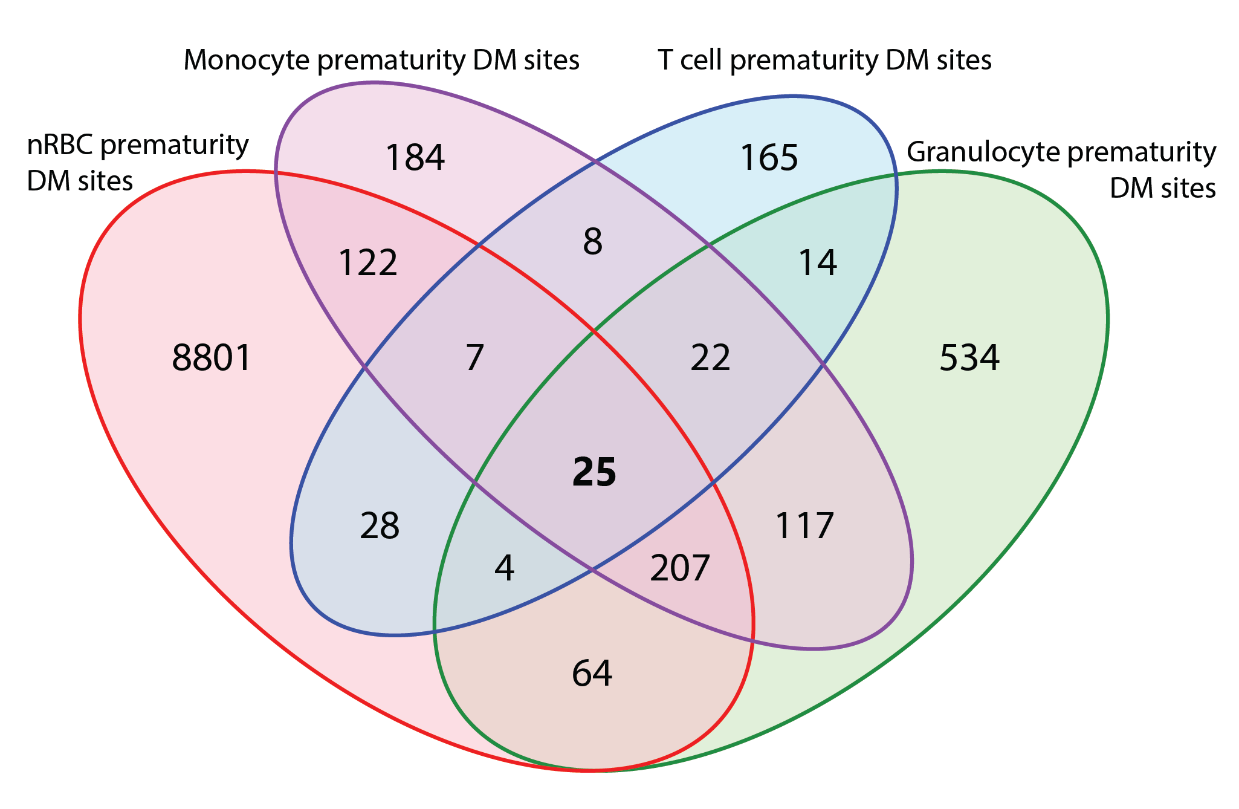


**Figure S2. Overlap of prematurity-DM sites between each hematopoietic cell type**. The 25 common prematurity-DM sites are shown in bold text.
